# Influence of Methylenetetrahydrofolate Reductase C677T and A1298C Polymorphism on High-Dose Methotrexate-Related Toxicities in Pediatric Non-Hodgkin Lymphoma Patients

**DOI:** 10.3389/fonc.2021.598226

**Published:** 2021-02-26

**Authors:** Suying Lu, Xiaoqin Zhu, Wei Li, Huimou Chen, Dalei Zhou, Zijun Zhen, Feifei Sun, Junting Huang, Jia Zhu, Juan Wang, Yizhuo Zhang, Xiaofei Sun

**Affiliations:** ^1^ State Key Laboratory of Oncology in South China, Collaborative Innovation Center for Cancer Medicine, Sun Yat-sen University Cancer Center, Guangzhou, China; ^2^ Department of Pediatric Oncology, Sun Yat-sen University Cancer Center, Guangzhou, China; ^3^ Department of Cardiology, Guangzhou Women and Children’s Medical Center, Guangzhou Medical University, Guangzhou, China; ^4^ Department of Molecular Diagnostics, Sun Yat-Sen University Cancer Center, Guangzhou, China

**Keywords:** MTHFR C677T, MTHFR A1298C, high-dose methotrexate, toxicity, pediatric patients, non-Hodgkin lymphoma

## Abstract

**Purpose:**

This retrospective study aimed to investigate the relationships between the methylenetetrahydrofolate reductase (MTHFR) C677T/A1298C and high-dose methotrexate (HD-MTX)-related toxicities in pediatric non-Hodgkin lymphoma (NHL) patients.

**Patients and Methods:**

We reviewed the medical records of 93 NHL patients aged under 18 years who received HD-MTX therapy at the dose of 5 g/m^2^ with 24-h infusion at Sun Yat-sen University Cancer Center between 2014 and 2019.

**Results:**

There were 61 males and 32 females, with a median age of 8.8 years (0.9–15.8 years). The tumor types included lymphoblastic lymphoma (n = 38), Burkitt’s lymphoma (n = 31), anaplastic large cell lymphoma (n = 18), diffuse large B-cell lymphoma (n = 6). Overall, 355 courses of HD-MTX therapy were prescribed. All patients were rescued with calcium folinate 12 h after the end of MTX infusion. We found that plasma MTX levels > 0.2 μmol/L at 48 h post-infusion increased the risk of developing oral mucositis (2.4% VS. 9.5%, P = 0.018). Also, patients carrying the C677T and T677T genotypes had tendencies to be more susceptible to oral mucositis (P = 0.034). Patients harboring mutant 677T allele were more likely to develop leucopenia (38.5 vs. 50.3%, P = 0.025) and thrombocytopenia (22.0 vs. 32.4%, P = 0.028). For polymorphism A1298C, the mutant genotype played a protective role in vomiting (11.1 vs. 4.3%, P = 0.018) but increased the risk of anemia (23.8 vs. 41.7%, P < 0.001) and leucopenia (38.1 vs. 50.3%, P = 0.021).

**Conclusion:**

Childhood NHL patients harboring C677T genotype were more vulnerable to oral mucositis, leucopenia, and thrombocytopenia, while those with A1298C genotype were at a decreased risk of vomiting and more likely to develop anemia and leucopenia.

## Introduction

Non-Hodgkin lymphoma (NHL), the fourth most common malignancy across the pediatric age spectrum, is a heterogeneous group of lymphoid malignancies (1, 2). In children, NHL comprises of four main categories, namely, lymphoblastic lymphoma (LBL), Burkitt lymphoma (BL), diffuse large B-cell lymphoma (DLBCL), and anaplastic large cell lymphoma (ALCL) (3). The current overall survival rate of pediatric NHL exceeds 80% due to dramatic progress in developing risk-adapted curative therapy (1), in which methotrexate (MTX) plays a crucial part.

MTX is a well-known folate analog that enters the cell *via* active transport mediated by the reduced folate carrier (RFC1). Subsequently, MTX arrests the folic acid cycle by inhibiting dihydrofolate reductase and influences other important enzymes involved in the folate pathway, such as methylenetetrahydrofolate reductase (MTHFR), an enzyme that interferes with nucleic acid synthesis and favors cell death (4, 5). The imbalance of folate homeostasis may cause DNA synthesis arrest and could disrupt DNA and protein methylation reaction (6). Thus, MTX is used to treat a variety of cancers (4–9). A high-dose MTX (HD-MTX) regimen, referred to the administration of a dosage ranging from 0.5 g/m^2^ to 12.0 g/m^2^ or even higher, is commonly used to treat childhood acute lymphoblastic leukemia (ALL), lymphoma and pediatric osteosarcoma (5, 10, 11). Despite its wide range of therapeutic efficacy, toxicities related to HD-MTX including reversible myelosuppression, nausea, vomiting, diarrhea, hepatotoxicity, nephrotoxicity, neurotoxicity, and particularly oral mucositis should not be neglected (4, 10–12). HD-MTX-related toxicities can not only lead to interruption or discontinuation of chemotherapy and increase relapse risk, but also affect the quality of life of patients. Therefore, identifying predictors of MTX toxicity is a key to determine effective individual dosage adjustment and to minimize adverse events (13).

**Figure 1:**
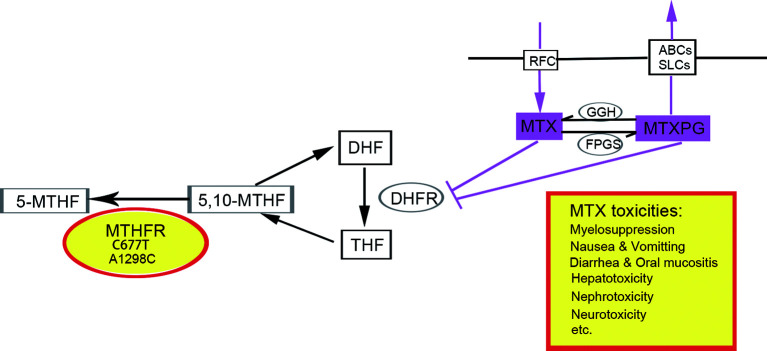
The role of MTHFR polymorphism in folate cycle and MTX metabolism. Polymorphisms in gene MTHFR could reduce the enzyme activity, leading to increased availability of 5,10-MTHF and decreased 5-MTHF. Consequently, the impaired conversion of 5, 10-MTHF to 5-MTHF could modify folate pools and in turn, alter the response of malignant and nonmalignant cells to MTX and potentially aggravates its toxicity. 5-MTHF, 5-methyltetrahydrofolate; 5,10-MTHF, 5,10-methylenetetrahydrofolate; DHF, dihydrofolate; THF, tetrahydrofolate; MTX, methotrexate; MTXPG, methotrexate polyglutamated forms. Transporters, RFC, reduced folate carrier; ABCs, ABC family transporters; SLCs, SLC family transporters. Enzymes, MTHFR, methylenetetrahydrofolate reductase; DHFR, dihydrofolate reductase; GGH, c-glutamyl hydrolase; FPGS, folylpolyglutamyl synthase.

The responses to MTX exhibit remarkable interindividual variability, making it difficult to predict who will develop more serious adverse events caused by HD-MTX (10, 12). In addition, accumulating pharmacogenetic studies have revealed that polymorphisms of enzymes involved in the folate pathway could lead to variability in response to MTX- and HD-MTX-related toxicities in various malignancies (12). For example, single nucleotide polymorphism (SNP) in the gene MTHFR, involved in MTX metabolism, demonstrated associations with a variety of nonhematologic and hematologic malignancies (10, 14, 15). MTHFR is a key enzyme for intracellular folate homeostasis and metabolism because it catalyzes the irreversible conversion of 5,10-methylenetetrahydrofolate (5,10-MTHF), essential for purine and thymidine synthesis, to 5-methyltetrahydrofolate (5-MTHF), essential for protein synthesis and nucleic acid methylation (4). The two most extensively studied SNPs of MTHFR in relation to the toxicities of MTX are the C677T variant (Ala222Val, rs1801133) and A1298C variant (Glu 429Ala, rs1801131), both dampening enzyme activity by 40–70% (4, 16) (shown in **Figure 1**).

Jared et al. reviewed the studies performed on MTX-induced toxicities across Asian and Caucasian pediatric and adult cancer patients for the MTHFR C677T and A1298C polymorphisms and observed controversial conclusions depending on the patient groups and subgroups investigated in the different systematic reviews as well as the genetic models utilized (7). Also, as most existing studies focused on ALL rather than NHL, there is limited evidence available on the role of C677T and A1298C polymorphisms in HD-MTX-related toxicities in pediatric NHL, with results varying considerably in different studies (10, 14, 16, 17). A multicenter trial NHL-BFM95 with 484 pediatric patients who received 4- or 24-h HD-MTX infusion regimens found that although LBL was significantly associated with MTHFR C677T genotype, this polymorphism did not appear to play a role in the therapy-associated toxicity (18). Noriko Shimasaki also concluded that no significant differences in the development of HD-MTX induced toxicity were observed for the different MTHFR C677T in children with NHL or ALL (19). In contrast, many published studies have suggested significant correlations between the C677T polymorphism and the risk of developing adverse events following MTX-therapy in patients with NHL, including hematologic and non-hematologic toxicity, especially mucositis (7). Very few published information is currently available on the influence of the MTHFR A1298C polymorphism on the development of HD-MTX-associated toxicities and are accompanied with controversial results. Therefore, the primary aim of this retrospective study was to evaluate the influence of C677T and A1298C polymorphisms on HD-MTX-related toxicities in children with NHL treated according to the modified NHL-BFM 95 protocol.

## Patients and Methods

### Patients and Treatment

We reviewed medical records of patients aged ≤ 18 years and diagnosed as NHL at the Sun Yat-sen University Cancer Center (SYSUCC) between 2014 and 2019. The patients were staged according to the new International Pediatric NHL Staging System (20). Only intermediate- and high-risk patients were included. This study was approved by the Institutional Review Board and the Research Ethics Committee of SYSUCC. The ethical approval batch number is B2019-231-01. Besides, this study was registered in the ClinicalTrials.gov and obtained the Clinical Trials. gov ID (NCT042839).

All enrolled patients received treatment according to the modified NHL-BFM95 protocol including MTX therapy at a dose of 5 g/m^2^. Each dose of HD-MTX therapy was followed by 6–7 times of calcium folinate (CF) rescue 12 h after the end of the MTX infusion, at a dose of 15 mg/m^2^ every 6 h. To maintain the urine pH at approximately 7–8, intravenous hydration and alkalization at the dose of 1,500 ml/m^2^ were achieved 12 h prior to the initiation of the HD-MTX administration (D0) and 3,000 ml/m^2^ per day lasted for the following 3 days (D1–D3). CF was also given from D1–D3 for mouth rinsing to prevent oral mucositis. We closely monitored the volume and pH of the patients’ urine from D0 to D4.

### HD-MTX-Related Toxicities

HD-MTX-related toxicities including hematological suppression, hepatotoxicity, nephrotoxicity, oral mucositis, vomiting, and diarrhea were detailly recorded after the MTX treatment until the next course of chemotherapy. Adverse events were graded according to the Common Terminology Criteria for Adverse Events version (CTCAE) 3.0. For analyses, toxicity grades were dichotomized as grade 0, and grade I or II versus III or IV.

### MTX Delayed Elimination

Various cutoff points were used to define the presence of clinically meaningfully delayed elimination in different studies (10, 13, 19, 21–23). According to our protocol, the plasma MTX levels were monitored at 0, 24, 48, and 72 h from the initiation of HD-MTX infusion. If the MTX concentration at 48 h was higher than 1 μmol/L, additional CF was performed. Aumente et al. proposed 0.2 μmol/L as a clinical cut-off value for MTX-related toxicities (24). In consensus with other reports (21), we recruited 0.2 μmol/L to define low or high MTX levels at 48 h post-treatment.

### Genotypic Polymorphism

**Figure 2:**
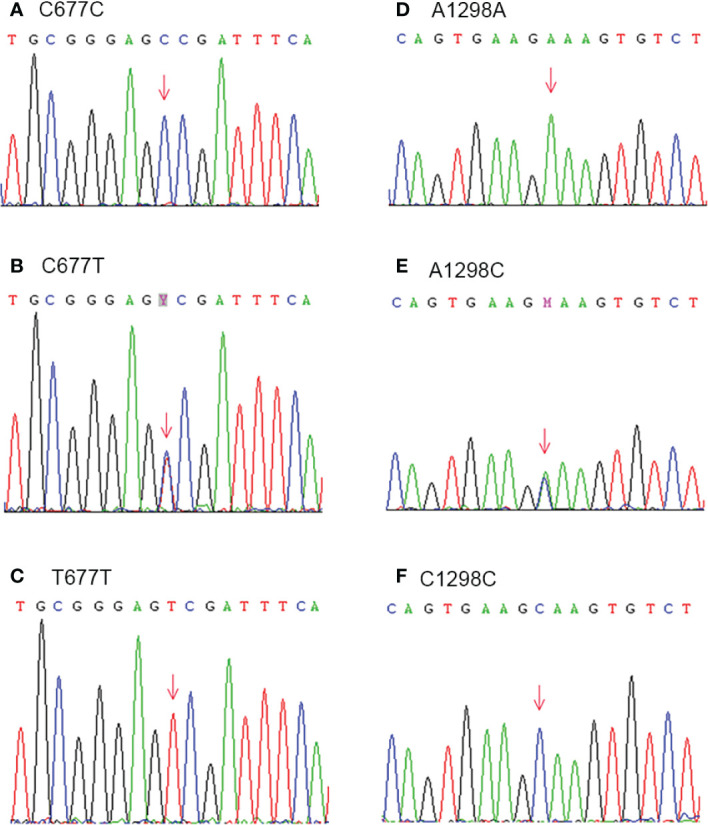
Genetic variations of MTHFR C677T (Ala 222 Val) **(A–C)** and A1298C (Glu 429 Ala) **(D–F)** were detected by PCR following Sanger sequencing.

The genetic variations of MTHFR were detected by PCR following Sanger sequencing. The PCR reaction was performed as follows: 94°C for 5 min; then 94°C 30 s, 58°C 30 s, 72°C 30 s for 32 cycles and finally, an extension at 72°C for 10 min. The lengths for MTHFR c.677C > T and c.1298 A > C were 246 bp and 256 bp, respectively. The primers were as following: for c.677C > T, Forward: 5′-TGCCCAGTCCCTGTGGTCTC-3′, Reverse: 5′-GGCAAGTGATGCCCATGTCG-3′; and for c.1298 A>C, Forward: 5′ TTTGGGGAGCTGAAGGACTA-3′, Reverse: 5′-ACAGGATGGGGAAGTCACAG-3′. The different genotypes are presented in **Figure 2**.

### Statistical Analyses

Statistical analyses were performed with IBM SPSS Statistics 25.0 software (SPSS Inc. Headquarters, 233 S. Wacker Drive, 11th floor, Chicago, Illinois 60606). Chi-square test was used to test deviations from the Hardy-Weinberg (H-W) equilibrium of MTHFR C677T/A1298C genotypes and analyze the associations between MTHFR polymorphism and toxicities. *P* values < 0.05 were considered significant. All data in our study have been put on record in Research Data Deposit (RDD) at SYSUCC for future reference and obtained the RDD number (RDDA2020001254, https://www.researchdata.org.cn).

## Results

### Clinical Characteristics of Patients

**Table 1** represents the clinical characteristics of the studied group. A total of 93 pediatric patients with the four NHL subtypes were included in this study. The median age of the study cohort was 8.8 years (range: 0.9–15.8 years). There were 61 (65.6%) males and 32 (34.4%) females. The majority of the patients were diagnosed as LBL (n = 38; 40.9%) and BL (n = 31; 33.3%), and the rest as ALCL (n = 18, 19.4%) and DLBCL (n = 6, 6.5%). Around two-thirds of the patients were diagnosed as stage IV (n = 60; 64.5.6%) and one third as stage III (n = 33; 35.5%) at the time of presentation. They were stratified into an intermediate- (n = 52; 55.9%) or high-risk (n = 41; 44.1%) group. Regarding the MTHFR polymorphism, both C677T and A1298T alleles exhibited the H-W equilibrium in the studied population (677C > T variant: *χ^2^* = 1.2224, *P* = 0.2689; 1298A > C variant: *χ^2^* = 0.0783, *P* = 0.7796). Half of the patients (47/93) carried the C677T wild-type genotype (CC), while 35/93 (37.63%) and 11/93 (11.83%) carried the heterozygous (CT) and homozygous (TT) variant genotype, respectively. As for the A1298C polymorphism, one patient failed to be genotyped. Wild-type (AA), heterozygous (CT) and homozygous (TT) variant genotype were observed in 49/93 (53.3%), 37/93 (40.2%) and 6/93 (6.5%) of the subjects, respectively. A total of 355 courses of HD-MTX therapy were analyzed in the present study. The plasma MTX level > 0.2 μmol/L at 48 h post-MTX infusion was observed in 63 (17.7%) courses.

**Table 1:** Clinical characteristics of the investigated pediatric patients with NHL (n = 93).

Characteristics	No. of patients, n (%)	No. of courses, n (%)
**Gender**		
Male	61 (65.6)	
Female	32 (34.4)	
**NHL subtypes**		
LBL	38 (40.9)	
BL	31 (33.3)	
ALCL	18 (19.4)	
DLBCL	6 (6.5)	
**Stage**		
III	33 (35.5)	
IV	60 (64.5)	
**Risk stratification**		
Intermediate	52 (55.9)	
High	41 (44.1)	
**MTHFR C677T (93/355)**		
CC	47 (50.5)	182 (51.3)
CT	35 (37.6)	137 (38.6)
TT	11 (11.8)	36 (10.1)
CT/TT	46 (49.5)	173 (48.7)
**MTHFR A1298C (92/352)**		
AA	49 (53.3)	189 (53.7)
AC	37 (40.2)	140 (38.9)
CC	6 (6.5)	23 (6.5)
AC/CC	43 (46.7)	163 (45.4)
**MTX plasma level at 48 h**		
≤ 0.2umol/L		292 (82.3)
> 0.2umol/L		63(17.7)

NHL, Non-Hodgkin Lymphoma; MTHFR, methylenetetrahydrofolate reductase; MTX, methotrexate; h, hour.

### The Role of Plasma MTX Levels in HD-MTX-Related Toxicity

The incidence rate of HD-MTX-related toxicities is shown in **Table 2**. The most frequently observed toxicity was myelosuppression. The results showed that grade III–IV leucopenia and neutropenia occurred in nearly half of the patients. However, the incidence of severe diarrhea (n = 0), nephrotoxicity (n = 0) and hepatotoxicity (n = 6) were too low to be further analyzed. A statistically significant difference was found comparing the incidence rate of oral mucositis in courses with low and high MTX level at 48 h (2.4 vs. 9.5%, *P* = 0.018, **Table 3**).

**Table 2:** Prevalence of HD-MTX-related toxicities in children with NHL.

Toxicity	Number of courses (%)	
	Grade I–II	Grade III–IV
Oral mucositis	66(18.6)	13(3.7)
Diarrhea	27(7.6)	0(0)
Vomiting	127(35.7)	30(8.4)
Nephrotoxicity	1(0.3)	0(0)
Hepatotoxicity	53(14.9)	6(1.7)
Anemia	182(51.3)	115(32.3)
Leucopenia	124(34.9)	157(44.3)
Neutropenia	83(23.3)	164(46.2)
Thrombocytopenia	38(10.7)	96(27.0)

HD-MTX, high-dose methotrexate.

**Table 3:** The relationships between MTX level at 48 h and HD-MTX-related toxicities.

Toxicity (GIII–IV) (No of courses)	MTX level at 48 h		*χ^2^*	*P*
	≤0.2 μmol/L (292)	>0.2 μmol/L (63)		
Oral mucositis (13)	7(2.4%)	6(9.5%)	5.577	0.018
Vomiting (30)	26(8.9%)	4(6.3%)	0.437	0.508
Anemia (115)	96(32.9%)	19(30.2%)	0.175	0.676
Leucopenia (157)	129(44.2%)	28(44.4%)	0.001	0.969
Neutropenia (164)	135(46.2%)	29(46.0%)	0.001	0.977
Thrombocytopenia (96)	80(27.4%)	16(25.4%)	0.105	0.746

### Associations Between Genetic Polymorphisms and Plasma MTX Levels

We also conducted Chi-square tests to assess the influence of genetic polymorphisms on plasma MTX levels. Plasma MTX levels at 48 h were found to be independent for both C677T and A1298C polymorphisms (**Table 4**). However, MTX levels at 72 h could not be analyzed due to a low occurrence rate of > 0.2 μmol/L.

**Table 4:** Associations between genetic polymorphisms and plasma MTX level at 48 h.

Genetic polymorphism (No of courses)	MTX level at 48 h		*χ^2^*	*P*
	≤0.2 μmol/L	>0.2 μmol/L		
**MTHFR C677T (355)**	292	63		
CC (182)	148(50.7%)	34(50.4%)	0.224	0.894
CT (137)	114(39.0%)	23(36.5%)
TT (36)	30(10.3%)	6(9.5%)
CT/TT (173)	144(49.3%)	29(46.0%)	0.224	0.636
**MTHFR A1298C (352)**	290	62		
AA (189)	157(54.1%)	32(51.6%)	1.275	0.529
AC (140)	114(39.3%)	26(41.9%)
CC (23)	19(6.6%)	4(6.5%)
AC/CC (163)	133(45.9%)	30(48.4%)	0.131	0.719

### Associations Between Genetic Polymorphisms and HD-MTX-Related Toxicities

The Chi-square test was used to analyze the associations between MTHFR C677T/A1298C polymorphism and the incidence rate of grade III-IV toxicities (**Table 5**). Polymorphism C677T was found to be significantly correlated with oral mucositis (*P* = 0.034), leucopenia (*P* = 0.025) and thrombocytopenia (*P* = 0.028). Polymorphism A1298C showed associations with vomiting (*P* = 0.018), anemia (*P* < 0.001), and leucopenia (*P* = 0.021).

**Table 5:** Associations between genetic polymorphisms and HD-MTX-related toxicities.

Toxicity (GIII–IV)	MTHFR C677T			*χ^2^*	*P*	MTHFR A1298C			*χ^2^*	*P*
(No. of courses)	CC(182)	CT(137)	TT(36)			AA(189)	AC(140)	CC(23)		
	CC(182)	CT/TT(173)				AA(189)	AC/CC(163)			
Oral mucositis (13)	4(2.2%)	5(3.6%)	4(11.1%)	6.768	0.034	4(2.1%)	8(5.7%)	1(4.3%)	2.957	0.228
	4(2.2%)	9(5.2%)		2.27	0.132	4(2.1%)	9(5.5%)		2.85	0.091
Vomiting (30)	14(7.7%)	11(8.0%)	5(13.9%)	1.543	0.462	21(11.1%)	7(5.0%)	0(0%)	6.229	0.044
	14(7.7%)	16(9.2%)		0.28	0.598	21(11.1%)	7(4.3%)		5.55	0.018
Anemia (115)	56(30.8%)	45(32.9%)	14(38.9%)	0.926	0.630	45(23.8%)	58(41.4%)	10(43.5%)	12.915	0.002
	56(30.8%)	59(34.1%)		0.45	0.502	45(23.8%)	68(41.7%)		12.9	0.0003
Leucopenia (157)	70(38.5%)	64(46.7%)	23(63.9%)	8.439	0.015	72(38.1%)	71(50.7%)	11(47.8%)	5.370	0.068
	70(38.5%)	87(50.3%)		5.03	0.025	72(38.1%)	82(50.3%)		5.3	0.021
Neutropenia (164)	78(42.9%)	65(47.4%)	21(58.3%)	3.036	0.219	83(43.9%)	69(49.3%)	10(43.5%)	0.998	0.607
	78(42.9%)	86(49.7)		1.68	0.195	83(43.9%)	79(48.5%)		0.73	0.393
Thrombocytopenia (96)	40(22.0%)	39(28.5%)	17(47.2%)	9.938	0.007	46(24.3%)	44(31.4%)	3(13.0%)	4.345	0.114
	40(22.0%)	56(32.4%)		4.86	0.028	46(24.3%)	47(28.8%)		0.91	0.34

## Discussion

### Comparisons of the Clinical Characteristics in Our Study to Previous Reports

Conflicting results are reported in relation to the role of polymorphisms C677T/A1298C in MTX adverse events in NHL (16, 17). The reported conflicting conclusions might result from substantial heterogeneity of the studied population as the MTX toxicity profile could be altered by ethnicity, MTX doses, co-administration of other anticancer agents, renal function, hydration and alkalization, and folinate rescue dosage regimens (10, 11). Unlike most studies including heterogeneous groups of patients, regardless of the type of hematologic malignancy and MTX-based chemotherapy protocols, our study minimized the chances of reporting and detection biases due to random sampling by enrolling a homogenous group of children with NHL treated according to the modified NHL-BFM 95 protocol in China over the past 5 years. Moreover, only patients who received MTX at the dose of 5 g/m^2^/24h were enrolled. Thus, the impact on toxicities generated from different doses could be avoided. The gender ratio was approximately 2:1, with more males than females. LBL and BL were observed more frequently than ALCL and DLBCL in Chinese children. Many retrospective analyses in other countries have reported similar clinical characteristics of studied population in pediatric NHL (25–28). Both C677T and A1298T alleles were in the H-W equilibrium with a similar mutant rate (49.5% for C677T and 46.7% for A1298C). Moreover, both genotype frequencies were consistent with those in previous reports (16, 29).

### The Role of Plasma MTX Levels in HD-MTX-Related Toxicity

Many studies have suggested that high plasma MTX concentration and prolonged exposure to high levels of MTX were linked to the development of toxicities (12). Accordingly, monitoring plasma MTX during HD-MTX therapy has become mandatory to individually adjust hydration, alkalization, and leucovorin rescue (11, 12). We found that patients with plasma MTX levels > 0.2 μmol/L at 48 h were more pronounced to develop oral mucositis (2.4 vs. 9.5%, *P* = 0.018, **Table 3**). The correlation between higher MTX levels and oral mucositis were also reported in other researches enrolling pediatric leukemia or lymphoma (19, 29) and osteosarcoma (5, 12, 23). One proposed mechanisms for MTX-related mucositis is that MTX may be secreted in the saliva, resulting in elevated direct mucosal toxicity, altered glutathione metabolism, and gastrointestinal microflora, and varied inflammatory responses by proinflammatory cytokines, together with folate metabolic pathway genes, contributing to mucositis (12). Therefore, possible hypothesis would be that patients with higher plasma MTX levels were expected to have higher MTX concentrations secreted into saliva, leading to increased risk of oral mucositis. Based on this assumption, measurements of the MTX plasma level remain vital for monitoring severe mucositis, which would be helpful in personalized management of oral mucositis. Herein, additional preventive strategies like reinforcement of mouth rinsing with CF might be necessary for patients with MTX level > 0.2 μmol/L at 48 h. However, in a study by Yun Jung Choi et al. who analyzed a total of 402 chemotherapy courses in 111 patients with primary central nervous system lymphoma(PCNSL), the authors concluded that MTX-induced oral mucositis occurred independently of the serum MTX level and that serum MTX concentration might not be a valid marker for predicting mucositis (10). A potential explanation for such inconsistence was the dose of MTX in the protocol for PCNSL was 3.5 g/m^2^, much lower than our protocol and the usage of CF for rescue was different.

### Associations Between Genetic Polymorphisms and Plasma MTX Levels

Studies on the correlation between polymorphism C677T and MTX levels were more extensive than A1298C. Regarding C677T, Nina Erculj showed that the mean MTX level was significantly higher in pediatric NHL patients of at least one MTHFR 677T allele (6). Previous reports had similar results in lymphoma and ALL (10, 22, 29). On the contrary, other studies indicated that no significant differences in the plasma MTX concentrations were found for the different MTHFR C677T genotype in lymphoma (19), which was in line with our results in regard to which we found that the plasma MTX levels at 48 h were independent in both C677T and A1298C polymorphisms (**Table 4**). However, the intensive rescue therapy in our protocol might have covered the influence of genetic polymorphisms on the plasma MTX levels at 48 h. Moreover, previous pharmacogenomic studies have shown that *MTHFR* was not the only one SNP that could influence the distribution, efficacy, and toxicities of MTX. Other SNPs including *FPGS*, *GGH*, *SLCO1B1*, and *ABCB1* also had similar effects on MTX-pharmacokinetics variability, which might partly account for the aforementioned inconsistence (30).

### Associations Between Genetic Polymorphisms and HD-MTX-Related Toxicities

#### MTHFR C677T

Patients carrying T alleles at MTHFR C677T were reported to experience interruptions in MTX treatment more frequently in childhood ALL or lymphoma (29, 31). In this analysis, we found that although MTHFR C677T/A1298C polymorphism did not significantly affect plasma MTX levels at 48 h, C677T was significantly correlated with oral mucositis (2.2 vs. 3.6 vs. 11.1%, *P* = 0.034, **Table 5**), leucopenia (38.5 vs. 50.3%, *P*=0.025, **Table 5**), and thrombocytopenia (22.0 vs. 32.4%, *P* = 0.028, **Table 5**) and that patients with C677T and T677T genotypes seemed to be more susceptible to those toxicities. The negative effects of polymorphism C677T might be explained by the decreased MTHFR activity. In the case of C677T, C nucleotide is substituted by T nucleotide at position 677, resulting in an amino acid exchange from alanine to valine in the respective amino acid sequence, which lowers the affinity of the enzyme for its cofactor, flavin adenine dinucleotide (5, 14). As a result, individuals in mutant status (C677T and T677T) exhibit 60 and 30% of the normal MTHFR activity, respectively (4, 5). The lowered MTHFR activity increases the availability of 5,10-MTHF but decreases that of 5-MTHF. Consequently, the impaired conversion of 5, 10-MTHF to 5-MTHF could modify folate pools and in turn, alter the response of malignant and nonmalignant cells to MTX and potentially aggravates its toxicity (4, 5, 16). Faganel et al. confirmed that MTX clearance decreased to 73.8% in in childhood ALL and malignant lymphoma with the MTHFR 677TT genotype (29). Therefore, patients carrying variant alleles might have an increased risk of developing higher intolerance to MTX (14).

The elimination of MTX mainly relies on kidney and liver. However, MTX could be accumulated in the third space or binds to protein, from which MTX clearance would be much slower (32). Therefore, in the case of oral mucositis, we hypothesized although the T allele at MTHFR C677T had no impact on the plasma MTX levels, it would ultimately lead to the delayed MTX elimination in the saliva, provoking higher risks of oral mucositis. However, Yun Jung Choi et al. demonstrated that the incidence of and oral mucositis requiring treatment was highest among patients with the wild type in CNS lymphoma (10). A possible reason might lie in the different criteria used for severe adverse events and different analytical methods utilized. Therefore, more attention should be paid to patients with T alleles. Similarly, the variant C allele might have impact on the MTX levels in the bone marrow rather than the blood, increasing the risk of anemia and thrombocytopenia.

Our results were in consensus with most existing studies. Mohammad et al. demonstrated that MTHFR 677C > T polymorphism was an independent marker for predicting MTX‐associated hematological toxicity in NHL (14). Angelo et al. showed that pediatric NHL patients harboring 677T allele had an approximately six-fold greater risk of developing hematological toxicity compared with wild-type carriers (9). Nina Erculj et al. reported that compared to patients with wild-type genotype, MTHFR 677T allele carrier had higher odds of leucopoenia and thrombocytopenia, probably through modulating MTX pharmacokinetics in pediatric NHL patients (6). Moreover, Barbara et al. implied that the MTHFR 677TT polymorphism was associated with an increased incidence of mucositis after HD-MTX treatment in lymphoma (29). Besides, Donato et al. revealed that adult NHL patients carrying 677TT genotype significantly increased the risk of developing mucositis and thrombocytopenia (17). Based on our clinical observations, for patients with MTHFR 677T allele, we guess reinforcement of mouth rinsing with CF could reduce the risk of developing oral mucositis and the preventive use of recombinant human granulocyte colony-stimulating factor (G-CSF) may decrease the incidence of leucopenia. However, further clinical research is necessary to find out whether CF and G-CSF could improve or reverse the effects of T alleles.

#### MTHFR A1298C

Regarding the influence of A1298C polymorphism on MTX toxicities, Nina Erculj et al. confirmed that MTHFR 1298A > C did not show any associations with myelosuppression, hepatotoxicity, nephrotoxicity, gastrointestinal toxicity and mucositis in pediatric NHL (6). However, other authors reported significant correlations between MTHFR A1298C and MTX toxicities but have not validated whether it plays a protective role against bone marrow and hepatic toxicity in children with ALL or NHL. Barbara et al. found that lymphoma patients homozygous for the variant MTHFR were at a decreased risk for leucopenia (29). Donato et al. demonstrated that NHL patients with 1298CC genotype were at a higher risk for developing mucositis (17). In a recent study by Goekkurt et al., the authors described that A1298C polymorphism seemed to be an independent predictor for hepatic toxicity with a higher risk for the 1298CC genotype with respect to AC and AA genotypes (33). Interestingly, unlike C677T, polymorphism A1298C was not suggested to increase the risk of oral mucositis but still correlated with hematological suppression in the present study. The mutant genotypes increased the risk of anemia (23.8 vs. 41.7%, *P* < 0.001, **Table 5**) and leucopenia (38.1 vs. 50.3%, *P* = 0.021, **Table 5**). However, the fact should not be neglected that the enzymatic activity of MTHFR is affected by A1298C polymorphism to a less extent than C677T, and that the remaining activity in the C1298C homozygous genotype still represents 60% of the normal status despite of the substitution of glutamate for alanine in the amino acid sequence (4, 14). Furthermore, our data indicated significant correlations between MTHFR A1298C polymorphism and vomiting. The mutant genotypes played a protective role in vomiting (11.1 vs. 4.3%, *P* = 0.018, **Table 5**). Intestinal toxicity is a common adverse event of MTX and may influence the entire intestinal tract, contributing to symptoms like nausea, bloating, and diarrhea. The gut microbiome has been reported to be able to modulate the host response to chemotherapeutic drugs and be associated with the intestinal toxicity of MTX (34, 35). We speculated that there might be an undiscovered positive role of C alleles at MTHFR A1298C on the MTX-gut microbiome interaction, relieving the symptomatic vomiting. Therefore, preventive administration of anti-vomiting agents would be more necessary for patients with wild-type genotype while G-CSF for patients with mutant genotype.

### Limitations

There are several limitations in this study. The major limitation is its retrospective nature. Additionally, MTX metabolism is influenced by not merely MTHFRC677T/A1298C but also many other genetic polymorphisms including SLC19A1, MTHFRG1793A (Arg594Gln) and ABCB1 (29, 36). Therefore, only taking MTHFRC677T/A1298C into consideration might not be convincing enough to explore their relationship with HD-MTX toxicities. Moreover, this is a single center study.

## Conclusion

Our results may provide a useful resource for clinicians to consider personalized medicine. Our data showed that patients with MTX levels higher than 0.2 μmol/L at 48 h were more vulnerable to oral mucositis. Analyzing MTHFR C677T and A1298C polymorphism prior to treatment might be useful for monitoring MTX-related adverse events in childhood NHL. Patients harboring mutant C677T genotype were more vulnerable to oral mucositis, leucopenia, and thrombocytopenia while those with mutant A1298C genotype were more likely to develop anemia and leucopenia but less susceptible to vomiting. Nevertheless, further prospective multicenter studies are required to validate the predictive significance of these associations.

## Data Availability Statement

The data sets presented in this study can be found in online repositories. The names of the repository/repositories and accession number(s) can be found below: www.researchdata.org.cn, RDDA2020001254.

## Ethics Statement

The studies involving human participants were reviewed and approved by the institutional review board and the research ethics committee of the Sun Yat-sen University Cancer Center (SYSUCC). Written informed consent from the participants’ legal guardian/next of kin was not required to participate in this study in accordance with the national legislation and the institutional requirements.

## Author Contributions

SL, XZ, WL, and HC contributed equally to this work in methodology, software, formal analysis, investigation, data curation, writing—original draft, and writing—review and editing. DZ, ZZ, FS, JH, JZ, and JW worked collaboratively in investigation and data curation. YZ and XS contributed equally to this work in conceptualization, supervision, and project administration. All authors contributed to the article and approved the submitted version.

## Funding

This research did not receive any specific grant from funding agencies in the public, commercial, or not-for-profit sector.

## Conflict of Interest

The authors declare that the research was conducted in the absence of any commercial or financial relationships that could be construed as a potential conflict of interest.

## References

[1] Minard-Colin V Brugieres L Reiter A Cairo MS Gross TG Woessmann W. Non-Hodgkin Lymphoma in Children and Adolescents: Progress Through Effective Collaboration, Current Knowledge, and Challenges Ahead. J Clin Oncol (2015) 33:2963–74. 10.1200/JCO.2014.59.5827 PMC4979194 26304908

[2] Gu X Zheng R Xia C Zeng H Zhang S Zou X. Interactions between life expectancy and the incidence and mortality rates of cancer in China: a population-based cluster analysis. Cancer Commun (Lond) (2018) 38:44. 10.1186/s40880-018-0308-x 29970165 PMC6029078

[3] Marginean CO Melit LE Horvath E Gozar H Chincesan MI. Non-Hodgkin lymphoma, diagnostic, and prognostic particularities in children - a series of case reports and a review of the literature (CARE compliant). Med (Baltimore) (2018) 97:e9802. 10.1097/MD.0000000000009802 PMC5842010 29465563

[4] Umerez M Gutierrez-Camino A Munoz-Maldonado C Martin-Guerrero I Garcia-Orad A. MTHFR polymorphisms in childhood acute lymphoblastic leukemia: influence on methotrexate therapy. Pharmgenomics Pers Med (2017) 10:69–78. 10.2147/PGPM.S107047 28392709 PMC5376125

[5] Lambrecht L Sleurs C Labarque V Dhooge C Uyttebroeck A. The role of the MTHFR C677T polymorphism in methotrexate-induced toxicity in pediatric osteosarcoma patients. Pharmacogenomics (2017) 18:787–95. 10.2217/pgs-2017-0013 28592186

[6] Erculj N Kotnik BF Debeljak M Jazbec J Dolzan V. The influence of folate pathway polymorphisms on high-dose methotrexate-related toxicity and survival in children with non-Hodgkin malignant lymphoma. Radiol Oncol (2014) 48:289–92. 10.2478/raon-2013-0076 PMC4110085 25177243

[7] Campbell JM Stephenson MD Bateman E Peters MD Keefe DM Bowen JM. Irinotecan-induced toxicity pharmacogenetics: an umbrella review of systematic reviews and meta-analyses. Pharmacogenomics J (2017) 17:21–8. 10.1038/tpj.2016.58 27503581

[8] Suthandiram S Gan GG Zain SM Bee PC Lian LH Chang KM. Effect of polymorphisms within methotrexate pathway genes on methotrexate toxicity and plasma levels in adults with hematological malignancies. Pharmacogenomics (2014) 15:1479–94. 10.2217/pgs.14.97 25303299

[9] D’Angelo V Ramaglia M Iannotta A Francese M Pota E Affinita MC. Influence of methylenetetrahydrofolate reductase gene polymorphisms on the outcome of pediatric patients with non-Hodgkin lymphoma treated with high-dose methotrexate. Leuk Lymphoma (2013) 54:2639–44. 10.3109/10428194.2013.784758 23488607

[10] Choi YJ Park H Lee JS Lee JY Kim S Kim TW. Methotrexate elimination and toxicity: MTHFR 677C>T polymorphism in patients with primary CNS lymphoma treated with high-dose methotrexate. Hematol Oncol (2017) 35:504–9. 10.1002/hon.2363 27781293

[11] Schmiegelow K. Advances in individual prediction of methotrexate toxicity: a review. Br J Haematol (2009) 146:489–503. 10.1111/j.1365-2141.2009.07765.x 19538530

[12] Park JA Shin HY. Influence of genetic polymorphisms in the folate pathway on toxicity after high-dose methotrexate treatment in pediatric osteosarcoma. Blood Res (2016) 51:50–7. 10.5045/br.2016.51.1.50 PMC4828529 27104192

[13] Liu SG Li ZG Cui L Gao C Li WJ Zhao XX. Effects of methylenetetrahydrofolate reductase gene polymorphisms on toxicities during consolidation therapy in pediatric acute lymphoblastic leukemia in a Chinese population. Leuk Lymphoma (2011) 52:1030–40. 10.3109/10428194.2011.563883 21534867

[14] Mashhadi MA Miri-Moghaddam E Arbabi N Bazi A Heidari Z Sepehri Z. C677T and A1298C polymorphisms of methylene tetrahydrofolate reductase in non-Hodgkin lymphoma: southeast Iran. Tumori (2018) 104:280–4. 10.5301/tj.5000634 28430351

[15] Lin S Yue J Guan X Yuan P Wang J Luo Y. Polymorphisms of MTHFR and TYMS predict capecitabine-induced hand-foot syndrome in patients with metastatic breast cancer. Cancer Commun (2019) 39:57. 10.1186/s40880-019-0399-z PMC6787984 31601265

[16] De Mattia E Toffoli G. C677T and A1298C MTHFR polymorphisms, a challenge for antifolate and fluoropyrimidine-based therapy personalisation. Eur J Cancer (2009) 45:1333–51. 10.1016/j.ejca.2008.12.004 19144510

[17] Gemmati D Ongaro A Tognazzo S Catozzi L Federici F Mauro E. Methylenetetrahydrofolate reductase C677T and A1298C gene variants in adult non-Hodgkin’s lymphoma patients: association with toxicity and survival. Haematologica (2007) 92:478–85. 10.3324/haematol.10587 17488658

[18] Seidemann K Book M Zimmermann M Meyer U Welte K Stanulla M. MTHFR 677 (C–>T) polymorphism is not relevant for prognosis or therapy-associated toxicity in pediatric NHL: results from 484 patients of multicenter trial NHL-BFM 95. Ann Hematol (2006) 85:291–300. 10.1007/s00277-005-0072-2 16463153

[19] Shimasaki N Mori T Samejima H Sato R Shimada H Yahagi N. Effects of methylenetetrahydrofolate reductase and reduced folate carrier 1 polymorphisms on high-dose methotrexate-induced toxicities in children with acute lymphoblastic leukemia or lymphoma. J Pediatr Hematol Oncol (2006) 28:64–8. 10.1097/01.mph.0000198269.61948.90 16462575

[20] Rosolen A Perkins SL Pinkerton CR Guillerman RP Sandlund JT Patte C. Revised International Pediatric Non-Hodgkin Lymphoma Staging System. J Clin Oncol (2015) 33:2112–8. 10.1200/JCO.2014.59.7203 PMC4461808 25940716

[21] Avivi I Zuckerman T Krivoy N Efrati E. Genetic polymorphisms predicting methotrexate blood levels and toxicity in adult non-Hodgkin lymphoma. Leuk Lymphoma (2014) 55:565–70. 10.3109/10428194.2013.789506 23829278

[22] Imanishi H Okamura N Yagi M Noro Y Moriya Y Nakamura T. Genetic polymorphisms associated with adverse events and elimination of methotrexate in childhood acute lymphoblastic leukemia and malignant lymphoma. J Hum Genet (2007) 52:166–71. 10.1007/s10038-006-0096-z 17180579

[23] Perez C Wang YM Sutow WW Herson J. Significance of the 48-hour plasma level in high-dose methotrexate regimens. Cancer Clin Trials (1978) 1:107–11. 316368

[24] Aumente D Buelga DS Lukas JC Gomez P Torres A Garcia MJ. Population pharmacokinetics of high-dose methotrexate in children with acute lymphoblastic leukaemia. Clin Pharmacokinet (2006) 45:1227–38. 10.2165/00003088-200645120-00007 17112298

[25] Burkhardt B Oschlies I Klapper W Zimmermann M Woessmann W Meinhardt A. Non-Hodgkin’s lymphoma in adolescents: experiences in 378 adolescent NHL patients treated according to pediatric NHL-BFM protocols. Leukemia (2011) 25:153–60. 10.1038/leu.2010.245 21030984

[26] Dokmanovic L Krstovski N Vukanic D Brasanac D Rodic P Cvetkovic M. Pediatric non-Hodgkin lymphoma: a retrospective 14-year experience with Berlin-Frankfurt-Munster (BFM) protocols from a tertiary care hospital in Serbia. Pediatr Hematol Oncol (2012) 29:109–18. 10.3109/08880018.2011.652342 22376014

[27] Gross TG Termuhlen AM. Pediatric non-Hodgkin’s lymphoma. Curr Oncol Rep (2007) 9:459–65. 10.1007/s11912-007-0064-6 17991353

[28] Muller J Csoka M Jakab Z Ponyi A Erlaky H Kovacs G. Treatment of pediatric non-Hodgkin lymphoma in Hungary: 15 years experience with NHL-BFM 90 and 95 protocols. Pediatr Blood Cancer (2008) 50:633–5. 10.1002/pbc.21144 17366531

[29] Faganel Kotnik B Grabnar I Bohanec Grabar P Dolzan V Jazbec J. Association of genetic polymorphism in the folate metabolic pathway with methotrexate pharmacokinetics and toxicity in childhood acute lymphoblastic leukaemia and malignant lymphoma. Eur J Clin Pharmacol (2011) 67:993–1006. 10.1007/s00228-011-1046-z 21509569

[30] Yang L Wu H de Winter B Sheng C Qiu H Cheng Y. Pharmacokinetics and pharmacogenetics of high-dose methotrexate in Chinese adult patients with non-Hodgkin lymphoma: a population analysis. Cancer Chemother Pharmacol (2020) 85:881–97. 10.1007/s00280-020-04058-4 32246190

[31] Shimasaki N Mori T Torii C Sato R Shimada H Tanigawara Y. Influence of MTHFR and RFC1 polymorphisms on toxicities during maintenance chemotherapy for childhood acute lymphoblastic leukemia or lymphoma. J Pediatr Hematol Oncol (2008) 30:347–52. 10.1097/MPH.0b013e318165b25d 18458567

[32] Walling J. From methotrexate to pemetrexed and beyond. A review of the pharmacodynamic and clinical properties of antifolates. Invest New Drugs (2006) 24:37–77. 10.1007/s10637-005-4541-1 16380836

[33] Goekkurt E Stoehlmacher J Stueber C Wolschke C Eiermann T Iacobelli S. Pharmacogenetic analysis of liver toxicity after busulfan/cyclophosphamide-based allogeneic hematopoietic stem cell transplantation. Anticancer Res (2007) 27:4377–80. 18214047

[34] Xinyi Huanga QF Raoa T Zhoua L Zenga X Tana Z Chen L. Leucovorin ameliorated methotrexate induced intestinal toxicity via modulation of the gut microbiota. Toxicol Appl Pharmacol (2020) 391:114900. 10.1016/j.taap.2020.114900 32061593

[35] Alexander JL Wilson ID Teare J Marchesi JR Nicholson JK Kinross JM. Gut microbiota modulation of chemotherapy efficacy and toxicity. Nat Rev Gastroenterol Hepatol (2017) 14:356–65. 10.1038/nrgastro.2017.20 28270698

[36] Nilsson TK Bottiger AK Henriquez P Serra Majem L. MTHFR polymorphisms and serum cobalamin affect plasma homocysteine concentrations differentially in females and males. Mol Med Rep (2014) 10:2706–12. 10.3892/mmr.2014.2521 25176448

